# Supplementary material for: TRPC3 is a major contributor to functional heterogeneity of cerebellar Purkinje cells
Source: eLife. 2019 Sep 5;8:e45590. doi: 10.7554/eLife.45590 (PMC6733575; doi:10.7554/eLife.45590)
Supplement: Supplementary file 1. [file elife-45590-supp1.docx]

**Table S1. Summary of the electrophysiological changes in gain- and loss-of-function TRPC3 mutants**

**Z- PCs**

| **Mouse line** | **In vitro** | | | | **In vivo** | | | | | | |
| --- | --- | --- | --- | --- | --- | --- | --- | --- | --- | --- | --- |
|  | *Cell-attach* | | | *Cur-inj* | *Simple spike* | | | *Complex spike* | | | CF-pause |
|  | FF | CV | CV2 | FF | FF | CV | CV2 | FF | CV | CV2 |  |
| **Gain-of-function** | ↑ | – | – | N/A | ↑ | ↑ | – | ↓ | ↑ | – | – |
| TRPC3^Mwk^ |  |  |  |  |  |  |  |  |  |  |  |
| **Loss-of-function** | ↓ | ↓ | ↓ | ↓ | ↓ | – | – | ↓ | – | – | ↑ |
| L7-TRPC3^KO^ |  |  |  |  |  |  |  |  |  |  |  |
| **Loss-of-function** | N/A | | | | ↓ | ↑ | ↑ | ↓ | ↑ | ↑ | ↑ |
| L7-TRPC3^KO^-EAAT4^eGFP^ |  |  |  |  |  |  |  |  |  |  |  |
| **loss-of-function** | N/A | | | | ↓ | – | – | – | – | – | – |
| L7-TRPC3^cKO^ |  |  |  |  |  |  |  |  |  |  |  |

**Z+ PCs**

| **Mouse line** | **In vitro** | | | | **In vivo** | | | | | | |
| --- | --- | --- | --- | --- | --- | --- | --- | --- | --- | --- | --- |
|  | *Cell-attach* | | | *Cur-inj* | *Simple spike* | | | *Complex spike* | | | CF-pause |
|  | FF | CV | CV2 | FF | FF | CV | CV2 | FF | CV | CV2 |  |
| **Gain-of-function** | – | – | – | N/A | – | ↑ | – | – | ↑ | – | – |
| TRPC3^Mwk^ |  |  |  |  |  |  |  |  |  |  |  |
| **Loss-of-function** | – | – | – | – | – | – | – | – | – | – | – |
| L7-TRPC3^KO^ |  |  |  |  |  |  |  |  |  |  |  |
| **Loss-of-function** | N/A | | | | – | – | – | ↓ | – | – | – |
| L7-TRPC3^KO^-EAAT4^eGFP^ |  |  |  |  |  |  |  |  |  |  |  |
| **loss-of-function** | N/A | | | | – | – | – | – | – | – | – |
| L7-TRPC3^cKO^ |  |  |  |  |  |  |  |  |  |  |  |

**Table S2. *In vitro* and *in vivo* electrophysiological kinetics**

**A.** *In vitro* cell-attached recording in L7-TRPC3^KO^ mice

| **L7-TRPC3^KO^** | | | | | |
| --- | --- | --- | --- | --- | --- |
| *Lobule I-III* | | | | | |
|  | Wild-type | L7-TRPC3^KO^ | t-value | df | P-value(t test) |
| Mice # | 5 | 6 | N/A | N/A | N/A |
| Cells # | 43 | 40 | N/A | N/A | N/A |
| Frequency (Hz) | 55.4±21.8 | 44.1±15.6 | 2.69 | 81 | 0.009 |
| CV | 0.08±0.03 | 0.07±0.03 | 2.19 | 81 | 0.031 |
| CV2 | 0.08±0.04 | 0.06±0.03 | 2.61 | 81 | 0.011 |
| *Lobule X* | | | | | |
|  | Wild-type | L7-TRPC3^KO^ | t-value | df | P-value(t test) |
| Mice # | 4 | 10 | N/A | N/A | N/A |
| Cells # | 35 | 36 | N/A | N/A | N/A |
| Frequency (Hz) | 30.8±11.7 | 28.5±9.3 | 0.937 | 64 | 0.352 |
| CV | 0.05±0.02 | 0.06±0.03 | -1.13 | 71 | 0.263 |
| CV2 | 0.04±0.01 | 0.04±0.02 | -0.977 | 67 | 0.332 |

Values are shown as mean±s.d.,.

**B.** *In vitro* cell-attached recording in TRPC3^Mwk^ mice

| **TRPC3^Mwk^** | | | | | |
| --- | --- | --- | --- | --- | --- |
| *Lobule I-III* | | | | | |
|  | Wild-type | TRPC3^Mwk^ | t-value | df | P-value(t test) |
| Mice # | 4 | 4 | N/A | N/A | N/A |
| Cells # | 15 | 15 | N/A | N/A | N/A |
| Frequency (Hz) | 58.4±19.4 | 84.5±36.2 | -2.47 | 28 | 0.020 |
| CV | 0.13±0.06 | 0.12±0.03 | 0.68 | 28 | 0.501 |
| CV2 | 0.13±0.07 | 0.10±0.04 | 1.43 | 21.5 | 0.168 |
| *Lobule X* | | | | | |
|  | Wild-type | TRPC3^Mwk^ | t-value | df | P-value(t test) |
| Mice # | 4 | 4 | N/A | N/A | N/A |
| Cells # | 12 | 13 | N/A | N/A | N/A |
| Frequency (Hz) | 39.7±21.5 | 36.7±13.0 | 0.419 | 17.9 | 0.680 |
| CV | 0.13±0.06 | 0.09±0.04 | 1.75 | 17.3 | 0.099 |
| CV2 | 0.08±0.05 | 0.06±0.02 | 1.62 | 23 | 0.119 |

Values are shown as mean±s.d.,

**C**. *In vitro* whole-cell recording in L7-TRPC3^KO^ mice

| **L7-TRPC3^KO^** | | | | | |
| --- | --- | --- | --- | --- | --- |
| *Lobule I-III* | | | | | |
|  | Widetype | L7-TRPC3^KO^ | t-value | df | P-value(t test) |
| Mice # | 5 | 5 | N/A | N/A | N/A |
| Cells # | 17 | 17 | N/A | N/A | N/A |
| Slope (Hz/100pA) | 19.2±1.1 | 16.0±1.0 | -2.20 | 32 | 0.035 |
| Holding current(pA) | 406±22 | 440±19 | 1.16 | 32 | 0.254 |
| Peak amplitude (mV) | 55.8±4.3 | 55.9±3.9 | 0.02 | 32 | 0.987 |
| Half-width (ms) | 0.21±0.02 | 0.18±0.01 | -1.10 | 32 | 0.279 |
| AHP (mV) | 4.37±0.39 | 3.53±0.55 | -1.24 | 32 | 0.223 |
| *Lobule X* | | | | | |
|  | Widetype | L7-TRPC3^KO^ | t-value | df | P-value(t test) |
| Mice # | 4 | 5 | N/A | N/A | N/A |
| Cells # | 12 | 12 | N/A | N/A | N/A |
| Slope (Hz/100pA) | 11.5±1.1 | 10.4±0.5 | -0.95 | 22 | 0.354 |
| Holding current(pA) | 422±38 | 391±28 | -0.66 | 22 | 0.515 |
| Peak amplitude (mV) | 43.5±1.8 | 48.5±2.4 | 1.67 | 22 | 0.109 |
| Half-width (ms) | 0.30±0.02 | 0.27±0.01 | -0.91 | 15 | 0.377 |
| AHP (mV) | 5.81±1.15 | 3.58±0.64 | -1.70 | 22 | 0.108 |

Values are shown as mean±s.e.m.,

**D**. *In vivo* extracellular recording in L7-TRPC3^KO^ mice

| **L7-TRPC3^KO^** | | | | | |
| --- | --- | --- | --- | --- | --- |
| **Lobule I-III** | | | | | |
|  | Wild-type | L7-TRPC3^KO^ | t-value | df | P-value(t test) |
| Mice # | 8 | 7 | N/A | N/A | N/A |
| Cells # | 26 | 30 | N/A | N/A | N/A |
|  |  |  |  |  |  |
| *Simple spike* |  |  |  |  |  |
| Frequency (Hz) | 88.5±17.4 | 74.4±18.6 | 2.88 | 54 | 0.006 |
| CV | 0.42±0.07 | 0.45±0.10 | -1.17 | 54 | 0.246 |
| CV2 | 0.40±0.06 | 0.42±0.08 | -1.03 | 54 | 0.310 |
|  |  |  |  |  |  |
| *Complex spike* |  |  |  |  |  |
| Frequency (Hz) | 1.41±0.35 | 1.16±0.39 | 2.50 | 54 | 0.016 |
| CV | 0.75±0.11 | 0.75±0.09 | -0.217 | 54 | 0.829 |
| CV2 | 0.82±0.07 | 0.82±0.09 | -0.109 | 54 | 0.914 |
|  |  |  |  |  |  |
| CF-pause(ms) | 17.3±4.7 | 21.4±8.3 | -2.33 | 47 | 0.024 |
| **Lobule X** | | | | | |
|  | Wild-type | L7-TRPC3^KO^ | t-value | df | P-value(t test) |
| Mice # | 6 | 8 | N/A | N/A | N/A |
| Cells # | 24 | 32 | N/A | N/A | N/A |
|  |  |  |  |  |  |
| *Simple spike* |  |  |  |  |  |
| Frequency (Hz) | 50.0±12.9 | 50.2±15.5 | -0.053 | 54 | 0.958 |
| CV | 0.31±0.06 | 0.30±0.06 | 0.371 | 54 | 0.712 |
| CV2 | 0.28±0.06 | 0.28±0.06 | 0.041 | 54 | 0.967 |
|  |  |  |  |  |  |
| *Complex spike* |  |  |  |  |  |
| Frequency (Hz) | 0.98±0.34 | 0.85±0.35 | 1.41 | 54 | 0.164 |
| CV | 0.67±0.13 | 0.71±0.13 | -1.21 | 54 | 0.233 |
| CV2 | 0.77±0.10 | 0.78±0.11 | -0.557 | 54 | 0.580 |
|  |  |  |  |  |  |
| CF-pause(ms) | 33.5±9.3 | 36.8±13.2 | -1.09 | 56 | 0.281 |

Values are shown as mean±s.d.,

**E**. *In vivo* extracellular recording in TRPC3^Mwk^ mice

| **TRPC3^Mwk^** | | | | | |
| --- | --- | --- | --- | --- | --- |
| **Lobule I-III** | | | | | |
|  | Wild-type | Trpc3^mwk^ | t-valu | df | p-value(t test) |
| Mice # | 6 | 7 | N/A | N/A | N/A |
| Cells # | 40 | 36 | N/A | N/A | N/A |
|  |  |  |  |  |  |
| *Simple spike* |  |  |  |  |  |
| Frequency (Hz) | 89.1±15.3 | 110±22.6 | -4.58 | 60 | 2.41×10^-5^ |
| CV | 0.43±0.07 | 0.55±0.11 | -5.62 | 60 | 5.37×10^-7^ |
| CV2 | 0.42±0.06 | 0.41±0.04 | 1.15 | 74 | 0.254 |
|  |  |  |  |  |  |
| *Complex spike* |  |  |  |  |  |
| Frequency (Hz) | 1.16±0.37 | 0.97±0.24 | 2.68 | 68 | 0.009 |
| CV | 0.76±0.14 | 0.88±0.15 | -3.71 | 74 | 4.05×10^-4^ |
| CV2 | 0.83±0.10 | 0.81±0.08 | 0.870 | 74 | 0.387 |
|  |  |  |  |  |  |
| CF-Pause (ms) | 18.5±3.8 | 19.0±5.8 | -0.420 | 51 | 0.676 |
| **Lobule X** | | | | | |
|  | Wild-type | Trpc3^mwk^ | t-value | df | p-value(t test) |
| Mice No. | 5 | 6 | N/A | N/A | N/A |
| Cell No. | 24 | 20 | N/A | N/A | N/A |
|  |  |  |  |  |  |
| *Simple spike* |  |  |  |  |  |
| Frequency (Hz) | 45.3±10.4 | 50.6±13.6 | -1.47 | 42 | 0.148 |
| CV | 0.34±0.08 | 0.73±0.26 | -6.22 | 22 | 2.8×10^-6^ |
| CV2 | 0.32±0.08 | 0.31±0.09 | 0.181 | 42 | 0.857 |
|  |  |  |  |  |  |
| *Complex spike* |  |  |  |  |  |
| Frequency (Hz) | 0.85±0.27 | 0.71±0.33 | 1.56 | 42 | 0.126 |
| CV | 0.70±0.13 | 0.88±0.27 | -2.71 | 26 | 0.012 |
| CV2 | 0.75±0.10 | 0.73±0.12 | 0.438 | 42 | 0.664 |
|  |  |  |  |  |  |
| CF-Pause (ms) | 33.1±8.1 | 38.1±19.0 | -1.14 | 28 | 0.263 |

Values are shown as mean±s.d.,

**F**. *In vivo* extracellular recording in L7-TRPC3^KO^-EAAT4^eGFP^ mice

| **L7-TRPC3^KO^-EAAT4^eGFP^** | | | | | |
| --- | --- | --- | --- | --- | --- |
| **Z- PCs (Lobule IV- VI)** | | | | | |
|  | Wild-type | L7-TRPC3^KO^-EAAT4^eGFP^ | t-value | df | P-value(t test) |
| Mice # | 2 | 3 | N/A | N/A | N/A |
| Cells # | 14 | 16 | N/A | N/A | N/A |
|  |  |  |  |  |  |
| *Simple spike* |  |  |  |  |  |
| Frequency (Hz) | 72.7±26.5 | 36.5±23.2 | 3.99 | 28 | 4.4×10^-4^ |
| CV | 0.44±0.10 | 0.55±0.16 | -2.27 | 28 | 0.031 |
| CV2 | 0.34±0.12 | 0.44±0.12 | -2.43 | 28 | 0.022 |
|  |  |  |  |  |  |
| *Complex spike* |  |  |  |  |  |
| Frequency (Hz) | 1.18±0.36 | 0.80±0.23 | 3.49 | 28 | 0.002 |
| CV | 0.74±0.11 | 0.85±0.16 | -2.12 | 28 | 0.043 |
| CV2 | 0.85±0.07 | 0.94±0.12 | -2.39 | 28 | 0.024 |
|  |  |  |  |  |  |
| CF-pause (ms) | 21.1±9.5 | 39.1±27.1 | -2.41 | 18 | 0.027 |
| **Z+ PCs (Lobule IV- VI)** | | | | | |
|  | Wild-type | L7-TRPC3^KO^-EAAT4^eGFP^ | t-value | df | P-value(t test) |
| Mice # | 2 | 3 | N/A | N/A | N/A |
| Cells # | 12 | 12 | N/A | N/A | N/A |
|  |  |  |  |  |  |
| *Simple spike* |  |  |  |  |  |
| Frequency (Hz) | 33.0±9.8 | 36.6±19.5 | -0.550 | 21 | 0.588 |
| CV | 0.56±0.26 | 0.47±0.22 | 0.873 | 21 | 0.393 |
| CV2 | 0.39±0.11 | 0.41±0.17 | -0.333 | 21 | 0.742 |
|  |  |  |  |  |  |
| *Complex spike* |  |  |  |  |  |
| Frequency (Hz) | 1.02±0.20 | 0.74±0.24 | 3.03 | 20 | 0.007 |
| CV | 0.70±0.05 | 0.65±0.10 | 1.44 | 20 | 0.165 |
| CV2 | 0.80±0.06 | 0.78±0.11 | 0.464 | 20 | 0.648 |
|  |  |  |  |  |  |
| CF-pause (ms) | 46.1±22.0 | 43.4±34.8 | 0.216 | 20 | 0.831 |

Values are shown as mean±s.d.,

**G**. *In vivo* extracellular recording in L7-TRPC3^cKO^ mice

| **L7-TRPC3^cKO^** | | | | | |
| --- | --- | --- | --- | --- | --- |
| **Lobule I-III** | | | | | |
|  | Wild-type | L7-TRPC3^cKO^ | t-value | df | P-value(t test) |
| Mice # | 4 | 4 | N/A | N/A | N/A |
| Cells # | 25 | 30 | N/A | N/A | N/A |
|  |  |  |  |  |  |
| *Simple spike* |  |  |  |  |  |
| Frequency (Hz) | 86.5±10.9 | 72.9±9.1 | 5.05 | 53 | 5.6×10^-6^ |
| CV | 0.40±0.06 | 0.40±0.07 | 0.221 | 53 | 0.826 |
| CV2 | 0.38±0.05 | 0.38±0.05 | -0.222 | 53 | 0.825 |
|  |  |  |  |  |  |
| *Complex spike* |  |  |  |  |  |
| Frequency (Hz) | 1.15±0.29 | 1.23±0.31 | -0.940 | 53 | 0.352 |
| CV | 0.78±0.16 | 0.73±0.09 | 1.51 | 53 | 0.136 |
| CV2 | 0.79±0.09 | 0.78±0.07 | 0.570 | 53 | 0.571 |
|  |  |  |  |  |  |
| CF-pause(ms) | 19.0±3.6 | 21.5±5.9 | -1.83 | 53 | 0.073 |
| **Lobule X** | | | | | |
|  | Wild-type | L7-TRPC3^cKO^ | t-value | df | P-value(t test) |
| Mice # | 3 | 4 | N/A | N/A | N/A |
| Cells # | 17 | 29 | N/A | N/A | N/A |
|  |  |  |  |  |  |
| *Simple spike* |  |  |  |  |  |
| Frequency (Hz) | 51.6±13.4 | 47.0±11.6 | 1.21 | 44 | 0.234 |
| CV | 0.31±0.05 | 0.33±0.06 | -1.09 | 44 | 0.283 |
| CV2 | 0.29±0.05 | 0.29±0.04 | 0.025 | 44 | 0.980 |
|  |  |  |  |  |  |
| *Complex spike* |  |  |  |  |  |
| Frequency (Hz) | 0.98±0.34 | 0.94±0.31 | 0.448 | 44 | 0.656 |
| CV | 0.67±0.11 | 0.68±0.08 | -0.020 | 44 | 0.984 |
| CV2 | 0.73±0.11 | 0.77±0.09 | -1.50 | 44 | 0.140 |
|  |  |  |  |  |  |
| CF-pause(ms) | 33.0±10.4 | 37.9±11.6 | -1.49 | 46 | 0.143 |

Values are shown as mean±s.d.,

**Table S3. Summary of behavioral kinetics**

**A**, Performance of compensatory eye movements recording

| Freq. | OKR | | VVOR | | VOR | |
| --- | --- | --- | --- | --- | --- | --- |
|  | WT | MUT | WT | MUT | WT | MUT |
| 0.1 | 0.90±0.02 | 0.91±0.03 | 0.94±0.02 | 0.95±0.03 | 0.33±0.03 | 0.27±0.05 |
| 0.2 | 0.87±0.03 | 0.85±0.03 | 0.94±0.02 | 0.96±0.03 | 0.40±0.04 | 0.34±0.05 |
| 0.4 | 0.67±0.03 | 0.70±0.05 | 0.99±0.01 | 0.96±0.04 | 0.64±0.03 | 0.56±0.06 |
| 0.6 | 0.44±0.03 | 0.48±0.02 | 1.01±0.02 | 0.98±0.03 | 0.77±0.03 | 0.72±0.05 |
| 0.8 | 0.28±0.02 | 0.29±0.02 | 1.02±0.02 | 0.94±0.04 | 0.87±0.03 | 0.79±0.04 |
| 1.0 | 0.24±0.01 | 0.25±0.01 | 0.99±0.02 | 0.98±0.02 | 0.93±0.04 | 0.88±0.05 |

**B**, Gain adaptation of compensatory eye movements recording

| Time (mins) | VOR gain decrease | | VOR gain increase | | Freq. | OKR gain increase | |
| --- | --- | --- | --- | --- | --- | --- | --- |
|  | WT | MUT | WT | MUT |  | WT | MUT |
| 0 | 0.79±0.03 | 0.72±0.05 | 1.00±0.00 | 1.00±0.00 | 0.1 | 0.87±0.03 | 0.89±0.04 |
| 5 | 0.64±0.03 | 0.58±0.03 | 1.03±0.06 | 1.11±0.06 | 0.2 | 086±0.03 | 0.84±0.04 |
| 10 | 0.58±0.04 | 0.53±0.05 | 1.11±0.07 | 1.23±0.07 | 0.4 | 0.85±0.03 | 0.80±0.05 |
| 15 | 0.50±0.05 | 0.48±0.03 | 1.17±0.06 | 1.21±0.07 | 0.6 | 0.83±0.02 | 0.81±0.05 |
| 20 | 0.52±0.03 | 0.50±0.03 | 1.30±0.08 | 1.24±0.09 | 0.8 | 0.80±0.03 | 0.82±0.05 |
| 25 | 0.49±0.03 | 0.45±0.04 | 1.25±0.08 | 1.32±0.09 | 1.0 | 0.72±0.03 | 0.75±0.04 |
| 30 | 0.45±0.04 | 0.43±0.05 |  |  |  |  |  |

**C**, Phase adaptation of compensatory eye movements recording

| Time (mins) | Day1 | | Day2 | | Day3 | | Day4 | | Day5 | |
| --- | --- | --- | --- | --- | --- | --- | --- | --- | --- | --- |
|  | WT | MUT | WT | MUT | WT | MUT | WT | MUT | WT | MUT |
| 0 | 13±1 | 17±1 | 16±2 | 16±1 | 21±2 | 20±2 | 31±5 | 36±7 | 64±13 | 77±10 |
| 5 | 13±1 | 15±1 | 15±2 | 19±2 | 31±7 | 33±7 | 67±15 | 57±13 | 127±18 | 110±15 |
| 10 | 14±2 | 17±2 | 20±2 | 24±3 | 56±13 | 48±10 | 79±14 | 90±19 | 128±14 | 133±17 |
| 15 | 16±2 | 18±2 | 22±3 | 28±4 | 59±13 | 58±10 | 81±16 | 85±16 | 142±12 | 135±14 |
| 20 | 17±2 | 18±2 | 22±3 | 28±4 | 64±13 | 54±10 | 93±17 | 106±16 | 142±9 | 144±13 |
| 25 | 15±2 | 17±2 | 23±4 | 30±6 | 69±14 | 72±14 | 108±15 | 111±16 | 132±13 | 151±14 |
| 30 | 15±3 | 20±4 | 32±6 | 30±4 | 69±14 | 61±11 | 103±14 | 104±17 | 147±8 | 153±11 |

**D**, Statistics (repeated measures ANOVA) for compensatory eye movements recording

| Paradigms | Mice # (WT/MUT) | F-value | P-value |
| --- | --- | --- | --- |
| OKR (gain / phase) | 12/10 | 0.211 / 0.396 | 0.651 / 0.537 |
| VVOR (gain / phase) | 12/10 | 0.240 /0.757 | 0.629 / 0.394 |
| VOR (gain / phase) | 12/10 | 2.082 /0.038 | 0.165 / 0.848 |
| OKR gain increase | 12/10 | 0.037 | 0.850 |
| VOR gain increase | 12/11 | 0.346 | 0.563 |
| VOR gain decrease | 13/11 | 0.764 | 0.392 |
| Phase reversal (Day 5) | 10/9 | 0.035 | 0.854 |

Values are shown as mean±s.e.m., MUT is referred to as L7-TRPC3^KO^, and WT is referred to as littermate controls.

**E**. Statistics of CR percentage for eyeblink conditioning

| **CR percentage** | | | | | |
| --- | --- | --- | --- | --- | --- |
| Session # | Wild-type (N=15) | L7-TRPC3^KO^(N=15) | t-value | df | p-value |
| 1 | 4.82±1.7 | 1.24±0.67 | 0.046 | 28 | 0.963 |
| 2 | 20.8±7.0 | 5.50±1.9 | 1.35 | 28 | 0.189 |
| 3 | 37.5±9.3 | 16.1±7.5 | 2.30 | 28 | 0.029 |
| 4 | 58.9±9.6 | 27.4±8.9 | 3.50 | 28 | 0.002 |
| 5 | 69.0±9.2 | 47.1±10.9 | 2.43 | 28 | 0.022 |
| 6 | 59.6±9.7 | 39.0±8.1 | 2.27 | 28 | 0.031 |
| 7 | 75.4±6.6 | 61.4±8.6 | 1.53 | 28 | 0.138 |
| 8 | 89.2±5.6 | 71.5±8.0 | 1.91 | 28 | 0.066 |
| 9 | 93.5±3.3 | 79.8±7.0 | 1.25 | 28 | 0.220 |
| 10 | 96.0±1.4 | 88.2±6.1 | 0.577 | 28 | 0.569 |
| 11 | 84.5±3.3 | 76.9±6.3 | 0.701 | 28 | 0.489 |
| 12 | 88.1±4.3 | 89.2±4.3 | -0.080 | 28 | 0.937 |
| 13 | 96.5±1.6 | 90.6±2.5 | 0.667 | 28 | 0.510 |
| 14 | 93.0±2.3 | 91.5±3.5 | 0.150 | 28 | 0.882 |
| 15 | 88.1±6.9 | 82.2±8.0 | 0.295 | 28 | 0.770 |

**F**, Statistics of fraction eyelid closure for eyeblink conditioning

| **Fraction eyelid closure** | | | | | |
| --- | --- | --- | --- | --- | --- |
| Session # | Wild-type(N=15) | L7-TRPC3^KO^(N=15) | t-value | df | p-value |
| 1 | 0.016±0.01 | 0.012±0.00 | -0.404 | 28 | 0.689 |
| 2 | 0.070±0.03 | 0.015±0.00 | 0.387 | 28 | 0.702 |
| 3 | 0.183±0.06 | 0.056±0.03 | 1.75 | 28 | 0.092 |
| 4 | 0.295±0.06 | 0.117±0.05 | 2.46 | 28 | 0.020 |
| 5 | 0.460±0.11 | 0.211±0.06 | 3.64 | 28 | 0.001 |
| 6 | 0.276±0.06 | 0.192±0.05 | 1.25 | 28 | 0.223 |
| 7 | 0.360±0.06 | 0.313±0.08 | 0.508 | 28 | 0.615 |
| 8 | 0.548±0.07 | 0.422±0.08 | 1.52 | 28 | 0.139 |
| 9 | 0.611±0.08 | 0.399±0.06 | 2.75 | 28 | 0.010 |
| 10 | 0.653±0.07 | 0.530±0.08 | 1.38 | 28 | 0.179 |
| 11 | 0.540±0.08 | 0.463±0.07 | 1.51 | 28 | 0.141 |
| 12 | 0.537±0.08 | 0.510±0.06 | 0.907 | 28 | 0.372 |
| 13 | 0.684±0.07 | 0.601±0.06 | 1.38 | 28 | 0.178 |
| 14 | 0.574±0.05 | 0.532±0.06 | 0.353 | 28 | 0.727 |
| 15 | 0.697±0.09 | 0.515±0.08 | 1.47 | 28 | 0.153 |

Values are shown as mean±s.e.m., P values were all FDR corrected for multiple comparisons, statistics was done by the linear mixed-effect model analysis.

**G**, Performance of Erasmus Ladder performance

| Session # | Back steps % | | Short steps % | | Long steps % | |
| --- | --- | --- | --- | --- | --- | --- |
|  | WT | MUT | WT | MUT | WT | MUT |
| 1 | 3.24±0.43 | 2.65±0.54 | 19.9±3.9 | 26.1±4.4 | 46.6±3.8 | 45.9±3.4 |
| 2 | 2.42±0.48 | 1.93±0.61 | 18.6±4.7 | 27.6±6.8 | 61.2±4.8 | 55.5±6.1 |
| 3 | 1.76±0.51 | 1.60±0.55 | 17.3±5.0 | 23.8±6.3 | 67.4±5.0 | 61.5±6.0 |
| 4 | 1.45±0.53 | 1.82±0.62 | 14.4±4.9 | 21.3±5.3 | 74.9±5.0 | 64.4±5.5 |
| 5 | 0.47±0.08 | 0.57±0.14 | 15.1±5.5 | 22.0±5.7 | 77.3±5.3 | 68.4±5.4 |

| Session # | Jump % | | Lower steps % | |
| --- | --- | --- | --- | --- |
|  | WT | MUT | WT | MUT |
| 1 | 3.75±0.51 | 3.03±0.68 | 10.5±1.1 | 9.3±1.1 |
| 2 | 3.86±0.67 | 2.48±0.81 | 5.2±0.8 | 4.7±0.9 |
| 3 | 3.23±0.80 | 2.84±0.70 | 3.8±0.5 | 3.7±0.7 |
| 4 | 2.69±0.74 | 2.34±0.68 | 2.2±0.3 | 3.7±0.7 |
| 5 | 1.56±0.28 | 1.69±0.30 | 2.0±0.2 | 2.8±0.5 |

**H**, Statistics (repeated measures ANOVA) for Erasmus Ladder performance

| Step types | Mice # (WT/MUT) | F-value | p-value |
| --- | --- | --- | --- |
| Back steps | 16/16 | 0.011 | 0.917 |
| Short steps | 16/16 | 0.980 | 0.330 |
| Long steps | 16/16 | 0.923 | 0.344 |
| Jump | 16/16 | 0.464 | 0.501 |
| Lower step | 16/16 | 0.012 | 0.913 |

Values are shown as mean±s.e.m., MUT is referred to as L7-TRPC3^KO^, and WT is referred to as littermate controls.
